# The Microbiome Modifies Manifestations of Hemophagocytic Lymphohistiocytosis in Perforin‐Deficient Mice

**DOI:** 10.1002/eji.202451061

**Published:** 2024-11-16

**Authors:** Jasmin Mann, Solveig Runge, Christoph Schell, Katja Gräwe, Gudrun Thoulass, Jessica Lao, Sandra Ammann, Sarah Grün, Christoph König, Sarah A. Berger, Benedikt Hild, Peter Aichele, Stephan P. Rosshart, Stephan Ehl

**Affiliations:** ^1^ Institute for Immunodeficiency Center for Chronic Immunodeficiency (CCI), Medical Center‐ University of Freiburg Faculty of Medicine University of Freiburg Freiburg Germany; ^2^ Faculty of Biology University of Freiburg Freiburg Germany; ^3^ Department of Medicine II, Medical Center‐ University of Freiburg, Faculty of Medicine University of Freiburg Freiburg Germany; ^4^ Department of Microbiome Research, University Hospital Erlangen Friedrich‐Alexander‐Universität Erlangen‐Nürnberg (FAU) Erlangen Germany; ^5^ Institute for Surgical Pathology, Medical Center‐ University of Freiburg, Faculty of Medicine University of Freiburg Freiburg Germany; ^6^ Department of Gastroenterology, Hepatology and Transplantation Medicine Medical Faculty University of Duisburg‐Essen Essen Germany

**Keywords:** hemophagocytic lymphohistiocytosis, microbiome, perforin‐deficiency, Wildling

## Abstract

Primary hemophagocytic lymphohistiocytosis (HLH) is a life‐threatening hyperinflammatory syndrome caused by inborn errors of cytotoxicity. Patients with biallelic *PRF1* null mutations (encoding perforin) usually develop excessive immune cell activation, hypercytokinemia, and life‐threatening immunopathology in the first 6 months of life, often without an apparent infectious trigger. In contrast, perforin‐deficient (PKO) mice only develop HLH after systemic infection with lymphocytic choriomeningitis virus (LCMV). We hypothesized that restricted microbe–immune cell interactions due to specific pathogen‐free (SPF) housing might explain the need for this specific viral trigger in PKO mice. To investigate the influence of a “wild” microbiome in PKO mice, we fostered PKO newborns with Wildling microbiota (‘PKO‐Wildlings’) and monitored them for signs of HLH. PKO‐Wildlings survived long‐term without spontaneous disease. Also, systemic infection with vaccinia virus did not reach the threshold of immune activation required to trigger HLH in PKO‐Wildlings. Interestingly, after infection with LCMV, PKO‐Wildlings developed an altered HLH pattern. This included lower IFN‐γ serum levels along with improved IFN‐γ‐driven anemia, but more elevated levels of IL‐17 and increased liver inflammation compared with PKO‐SPF mice. Thus, wild microbiota alone is not sufficient to trigger HLH in PKO mice, but host–microbe interactions shape inflammatory cytokine patterns, thereby influencing manifestations of HLH immunopathology.

## Graphical abstract

This study investigates the microbiome as a potential factor underlying the apparent differences in immune stimulation required for HLH induction in perforin‐deficient mice compared to FHL‐2 patients. Using PKO‐Wildlings, we show that while immune activation by natural microbiota is not sufficient to induce HLH, it can influence manifestations of LCMV‐induced disease.

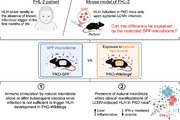

**Abbreviations**
CTLs: cytotoxic T lymphocytes
GPT: glutamate‐pyruvate transaminase
HLH: hemophagocytic lymphohistiocytosis
LCMV: lymphocytic choriomeningitis virus
PKO: perforin‐deficient
PKO‐SPF: PKO mice housed under SPF conditions
PKO‐Wildling: PKO mice with Wildling microbiome
sCD25: soluble interleukin‐2 receptor alpha chain
SPF: specific pathogen‐free
VV_WR_
: vaccinia virus strain WR

## 1 Introduction

Patients with inborn errors of lymphocyte cytotoxicity have a genetic predisposition to develop primary hemophagocytic lymphohistiocytosis (HLH)‐ a fatal syndrome of immune dysregulation mediated by hyperstimulated T cells and macrophages. Insufficient cytotoxicity of natural killer cells and T cells is caused by gene defects in *PRF1* (encoding perforin) or genes linked to lytic granule exocytosis [1, 2, 3, 4]. The clinical diagnosis of HLH relies on the fulfillment of at least five of eight clinical and laboratory criteria defined by the HLH‐study group [5]. Clinical manifestations encompass persistent fever, hepatosplenomegaly, and pancytopenia. Frequent additional symptoms are liver dysfunction and neurological symptoms [6, 7, 8]. Characteristic laboratory values include high levels of ferritin, hypertriglyceridemia and/or hypofibrinogenemia, and elevated soluble interleukin‐2 receptor alpha chain (sCD25) levels [5, 6]. Patients often have impaired natural killer cell cytotoxicity and hemophagocytosis is commonly observed in bone marrow, lymph nodes, spleen, liver, and cerebral spinal fluid [6, 9].

The current understanding of primary HLH pathophysiology largely stems from studies in perforin‐deficient (PKO) mice: Absent lymphocyte cytotoxicity impairs the timely removal of infected cells including antigen‐presenting cells, resulting in hyperstimulated CD8^+^ T cells [10]. IFN‐γ, predominantly produced by cytotoxic CD8^+^ T lymphocytes (CTLs), activates macrophages and prompts massive inflammatory cytokine release [7, 11, 12]. Tissue infiltration by activated T cells and macrophages, combined with hypercytokinemia, leads to the cardinal symptoms of HLH and, without intervention, to lethal multiorgan failure [6, 12].

Patients with perforin null mutations usually present with HLH in the first 6 (‐12) months of life [13, 14]. In some infants (<20%), the disease manifests in the context of viral infections, but in most patients, no obvious specific pathogenic trigger can be identified [7, 13, 15]. On the contrary, PKO mice do not develop HLH unless they are infected with lymphocytic choriomeningitis virus (LCMV) or murine cytomegalovirus (MCMV) [11, 16]. Systemic infections with these viruses are not controlled in the absence of perforin and induce a fatal disease resembling human primary HLH [11, 16]. Other viruses such as respiratory syncytial virus [17], pneumonia virus of mice [18], influenza virus [19], and vaccinia virus‐WR (VV_WR_) [20], all of which are controlled independent of perforin, fail to induce HLH [20, 21]. This observation indicates that the nature and extent of immune stimulation required for HLH induction varies between mice and humans. However, the underlying reason for this difference remains unknown.

In addition to interspecies and genetic variances, a notable difference between humans and laboratory animals, like PKO mice, is their restricted microbiome due to specific pathogen‐free (SPF) housing, depriving them of tonic immune stimulatory signals [22, 23, 24, 25]. To bridge this gap, the Wildling mouse model was recently established by a transfer of C57BL/6 (B6) embryos into pseudo‐pregnant wild mice [26]. B6‐Wildlings exhibit a stable and diverse microbiome closely resembling that of wild mice. Importantly, in two preclinical studies, they more accurately predicted the human immune response than SPF mice [26]. The increased translational value of Wildlings may be attributed to the influence of the microbiome on the fine‐tuning of the immune system, including modulation of T cell differentiation and the licensing of plasmacytoid dendritic cells (DC) for efficient T‐cell priming during pathogen encounters [27, 28, 29]. Thus, we hypothesized that introducing the Wildling microbiome into PKO mice might heighten the tonic activation status of the immune system and consequently, lower the threshold for HLH induction or even initiate spontaneous disease in the absence of an additional trigger. To test this hypothesis, we generated PKO‐Wildlings and closely monitored them for HLH parameters under resting conditions and after systemic viral infections.

## 2 Results

### 2.1 Reconstituting PKO Mice With Wildling Microbiota Does Not Provoke Spontaneous HLH

**FIGURE 1:**
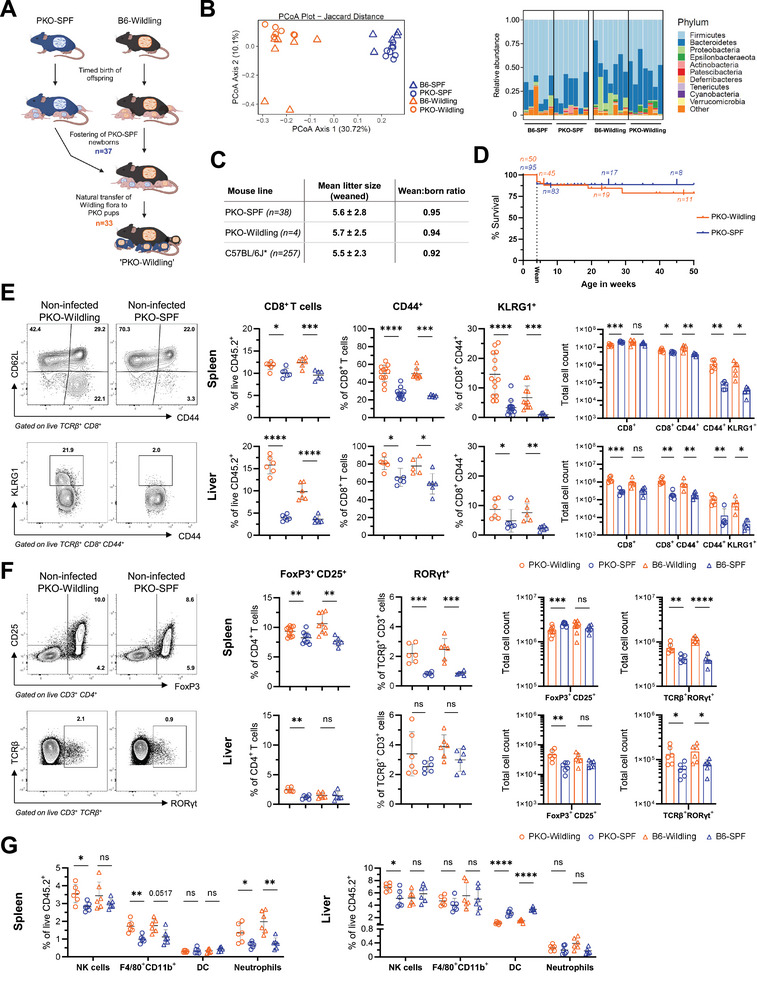
Transfer of a diverse microbiome into PKO mice does not provoke spontaneous HLH. (A) Experimental setup for generating PKO‐Wildlings. (B) Microbiome analysis of fecal samples from Wildlings and SPF mice. Principal coordinate analysis (PCoA) with distance calculation using Bray‐Curtis dissimilarity (left). Relative abundance at the rank of phylum (right). (C) Mean litter size and wean‐to‐born ratio for PKO‐Wildling, PKO‐SPF, and C57BL/6J mice [31]. (D) Kaplan–Meier curve of cumulative survival probability for PKO‐Wildling and PKO‐SPF mice at indicated time points after birth (*n* = number of mice). Ticks indicate censored animals. (E) Representative contour plots of indicated antigens on isolated splenocytes (left). Relative and absolute abundance of CD8^+^ T cells, CD44^+^, and KLRG1^+^ antigen‐experienced CD8^+^ T cells in the spleen and liver (right). (F) Contour plots depicting FoxP3, CD25, and RORγt expression (left). Frequency and total counts of FoxP3^+^ CD25^+^ CD4^+^ T_regs_ and RORγt^+^ T cells. (G) Abundance of indicated populations in the spleen and liver. (E–G) Data represent mean ± SD, pooled from at least two independent experiments with 3 mice/group, aged 9–16 weeks of age. Statistical differences were determined using unpaired Student's *t*‐test, Mann–Whitney test, or multiple unpaired *t*‐tests with correction for multiple comparisons using the Holm–Šidák method.

To investigate whether a diverse microbiome disrupts immune homeostasis in the absence of perforin resulting in spontaneous HLH, we generated PKO‐Wildlings by fostering PKO‐SPF newborns to female B6‐Wildlings that had just given birth to a litter [26]. Of the 37 PKO pups fostered by B6‐Wildling mothers, 33 survived until weaning (Figure 1A). Using this approach, PKO mice naturally acquired the Wildling microbiota early in life. [30]. To validate the successful colonization of fostered PKO mice with Wildling microbiome, we performed fecal 16S ribosomal gene profiling at 10 weeks of age. Principal coordinate analysis demonstrates clustering of PKO‐Wildlings with B6‐Wildlings, while PKO‐SPF and B6‐SPF mice formed a separate cluster (Figure 1B; Figure. S1A). Additionally, consistent with previous data [26, 30], taxon abundance analysis showed an increase in Bacteroidetes and a decrease in Firmicutes in both B6‐Wildlings and PKO‐Wildlings (Figure 1B, Figure S1A). Using the fostered offspring, we established a small colony of PKO‐Wildlings by homozygous breeding. PKO‐Wildlings had normal litter size and the wean‐to‐born ratio was comparable to PKO‐SPF and B6‐SPF mice (Figure 1C, [31]). There were no signs of systemic illness and survival of PKO‐Wildlings was similar to PKO‐SPF mice until 50 weeks of age (Figure 1D).

HLH diagnostic parameters were absent in a total of 8 PKO‐Wildlings sampled at different ages between 6 and 44 weeks (data not shown). Notably, we observed signs of immune alteration in lymphatic organs and the liver of Wildlings, as anticipated in a situation of increased microbial exposure [23, 27, 30, 32]. This included an expansion of the CD8^+^ T cell compartment, along with elevated proportions and total counts of antigen‐experienced (CD44^+^ CD62L^+/−^) and terminally differentiated (KLRG1^+^) CD8^+^ T cells in the spleen and liver of PKO‐Wildlings and B6‐Wildlings compared with PKO‐SPF and B6‐SPF mice (Figure 1E, gating strategy Figure S1B). In addition, PKO‐Wildlings showed higher proportions but lower absolute counts of CD4^+^ regulatory T cells (T_regs_) in the spleen, whereas in the liver, T_regs_ were increased (Figure 1F, gating strategy Figure S1C). IL‐17‐producing TCRαβ^+^ T cells, identified by staining of the retinoic acid‐related orphan receptor gamma t (RORγt), showed an increased frequency and total number, predominantly in the spleen and to a lesser extent in the liver, when comparing Wildling to SPF mice (Figure 1F, gating strategy Figure S1D). F4/80^+^ CD11b^+^ macrophages and neutrophils were elevated in the spleen of Wildlings, while there were no significant differences in the liver (Figure 1G, gating strategy Figure S1E). Comparable immunological alterations were observed in the mesenteric lymph nodes (Figure S1F). Overall, these findings suggest that the microbiome was efficiently transferred to fostered PKO animals and that diverse microbial exposure induces basal immune activation, but does not cause spontaneous disease in the absence of perforin.

### 2.2 Presence of Wildling Microbiome Does Not Lower the Immune Threshold for HLH Disease Induction

**FIGURE 2:**
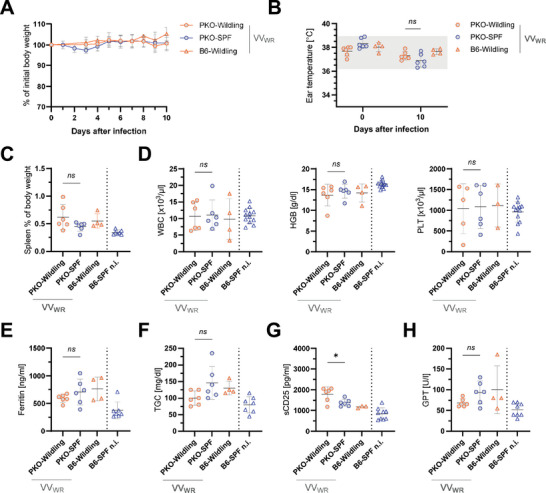
PKO‐Wildlings do not develop HLH after infection with vaccinia virus. PKO‐Wildling, B6‐Wildling, and PKO‐SPF mice were infected with 2 × 10^6^ pfu vaccinia virus WR (VV_WR_) and HLH parameters were analyzed 10 days later. (A) Body weight is shown as mean percentage ± SD of initial body weight pooled from 2 independent experiments with 2–3 mice/group. (B) Ear temperature on day 0 and day 10 after infection. The grey area represents the reference range of noninfected B6‐SPF animals (mean ± 2xSD). (C) Spleen weight as a percentage of body weight. (D) Number of white blood cells (WBC), hemoglobin (HGB) and platelet (PLT) count measured in EDTA‐blood. (E–H) Ferritin, triglycerides (TGC), soluble CD25 (sCD25), and glutamate‐pyruvate transaminase (GPT) in serum. (B–H) Symbols represent individual mice and shown is mean ± SD. Data were pooled from two independent experiments with 2–3 mice/group. Noninfected (n.i.) B6‐SPF mice are shown as a healthy reference. Statistical testing was performed using an unpaired Student's *t*‐test.

**FIGURE 3:**
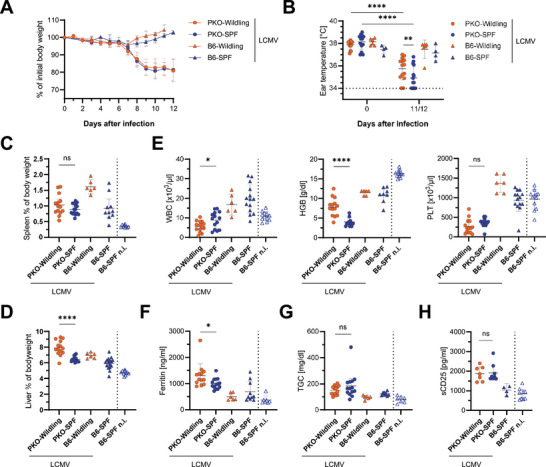
PKO‐Wildlings develop lethal HLH immunopathology after LCMV infection similar to PKO‐SPF mice. Mice were infected with 200 pfu LCMV‐WE i.v. and HLH parameters were assessed on day 11/12 after infection. (A) Body weight of LCMV‐infected mice at indicated time points. Shown is mean ± SD of 4 independent experiments with 2–4 mice/group. (B) Ear temperature on day 0 and day of analysis. (C, D) Spleen and liver weight as a percentage of body weight. (E) White blood cell (WBC) count, hemoglobin (HGB), and platelet numbers (PLT) measured in EDTA blood. (F–H) Ferritin, triglycerides (TGC), and soluble CD25 (sCD25) in serum of LCMV‐infected mice. (B–H) Symbols represent individual mice, shown as mean ± SD, pooled from 2–4 independent experiments with 2–4 mice/group. Healthy noninfected (n.i.) B6‐SPF mice are shown for comparison. Statistical testing to compare PKO‐Wildling and PKO‐SPF mice was done by two‐way ANOVA and Šidák post hoc analysis (B), Mann–Whitney test (C–E, G‐H), and unpaired Student's *t*‐test (B, F).

Next, we hypothesized that the Wildling microbiome might lower the threshold for initiating HLH, allowing pathogens that would not trigger HLH in PKO‐SPF mice to induce the disease. To test this hypothesis, we chose vaccinia virus (VV_WR_), which elicits a strong systemic T cell response, but virus elimination ultimately occurs without significant contribution of perforin [20]. However, PKO‐Wildlings did not develop lethal HLH after VV_WR_ infection. Weight loss and a drop in ear temperature, as usually observed in active HLH (see Figure 3A,B), were absent in PKO‐Wildling and PKO‐SPF mice on day 10 after infection (Figure 2A,B). Moderate infection‐associated splenomegaly was observed in VV_WR_‐infected mice in comparison to noninfected B6‐SPF mice (Figure 2C). Additional features typically observed in HLH, including pancytopenia and elevated levels of ferritin and triglycerides were absent or moderately increased in infected animals (Figure 2D–F). Levels of sCD25 were increased in PKO‐Wildling and PKO‐SPF animals in comparison to noninfected B6‐SPF mice and slightly higher in PKO‐Wildlings compared to PKO‐SPF (Figure 2G). The level of glutamate‐pyruvate transaminase (GPT) was slightly increased in VV_WR_‐infected mice compared to noninfected B6‐SPF mice (Figure 2H). Overall, VV_WR_ infection induced immune activation but did not lead to HLH in PKO‐Wildlings. This indicates that immune pre‐activation by the Wildling microbiome did not allow this virus to reach the immune activation threshold required for HLH induction.

### 2.3 PKO‐Wildlings Develop Fatal HLH After LCMV Infection

Primary HLH is a cytokine‐driven disease and it has been shown that the microbiome profoundly influences the cytokine response and outcome of influenza virus infection [24, 33]. We therefore investigated whether the Wildling microbiome impacts the outcome of LCMV‐induced HLH in PKO mice. Both PKO‐Wildling and PKO‐SPF mice started to lose body weight on day 8 after LCMV infection, reaching a mean loss of approximately 20% by day 12 (Figure 3A), and presented with decreased activity, ruffled fur, and hunched posture. Ear temperature was lower in PKO mice compared with controls and slightly higher in PKO‐Wildling than in PKO‐SPF mice (Figure 3B). Splenomegaly was observed in both PKO‐Wildling and PKO‐SPF mice (Figure 3C), while livers were significantly larger in PKO‐Wildlings (Figure 3D). Pancytopenia was evident in PKO‐Wildling and PKO‐SPF mice (Figure 3E). However, anemia was significantly less pronounced in PKO‐Wildling compared with PKO‐SPF mice (Figure 3E). Ferritin levels were slightly higher in PKO‐Wildlings, while triglycerides and sCD25 were similarly elevated in both groups (Figure 3F–H). Thus, although the presence of a wild microbiome had no impact on the number of clinical HLH criteria fulfilled in LCMV‐infected PKO mice or the severity of the disease, relevant differences were observed in the pattern of disease manifestations.

### 2.4 PKO‐Wildlings Present With Enhanced Liver Inflammation During Acute HLH

**FIGURE 4:**
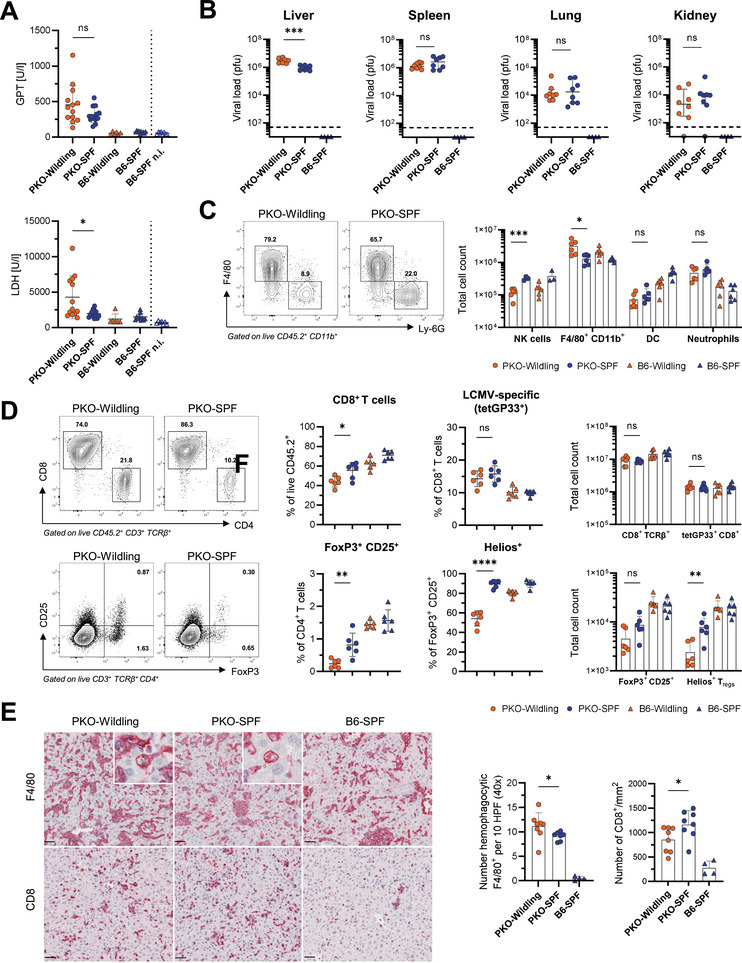
Presence of Wildling microbiota in PKO mice promotes liver pathology during LCMV‐induced HLH. (A) Glutamate‐pyruvate transaminase (GPT) and lactate‐dehydrogenase (LDH) in serum of LCMV‐infected mice analyzed on day 11/12 after infection. Noninfected (n.i.) B6‐SPF mice are shown as a reference. (B) Viral titers in the liver, spleen, lung, and kidney. Shown is the median with an interquartile range. (C) The contour plot depicts the staining of F4/80 and Ly‐6G on density‐isolated immune cells from the liver of LCMV‐infected mice (left). The graph shows absolute numbers of NK cells (NK1.1^+^ CD3^−^), F4/80^+^ CD11b^+^ macrophages, dendritic cells (DC, CD11c^+^ SiglecH^+^/CD11c^+^ MHCII^+^), and Neutrophils (CD11b^+^ Ly‐6G^+^) in the liver (right). (D) Representative flow cytometry plots showing expression of CD8, CD4, FoxP3, and CD25 on liver immune cells (left), and percentages and total numbers of indicated cell subsets (right). (E) Representative images of CD8 and F4/80 immunohistochemistry staining of liver sections with H&E counterstaining (scale bar = 50 µm). Insert: Examples of hemophagocytosis. Quantification of hemophagocytic macrophages per 10 high‐power fields (HPF) assessed based on F4/80 staining and number of CD8^+^ cells per mm^2^ liver tissue. (A, C–E) Shown is mean ± SD. Symbols represent individual mice pooled from 2–4 independent experiments with 2–4 mice/group. Statistical testing was performed using unpaired Student‘s *t*‐test (A), Mann–Whitney test (A, B, D, E), and multiple *t*‐tests with correction for multiple comparisons by Holm‐Šidák (C).

Considering the role of gut microbiota in modulating hepatic immune cell responses and inflammatory liver conditions [34], along with the pronounced hepatomegaly in observed PKO‐Wildlings, we specifically analyzed the influence of the Wildling microbiome on liver immunopathology during LCMV infection. The levels of GPT and lactate‐dehydrogenase were slightly increased in infected PKO‐Wildlings compared to PKO‐SPF mice, indicating more severe liver damage (Figure 4A). Furthermore, viral titers in the livers of PKO‐Wildlings were significantly higher than in the livers of PKO‐SPF mice, while they were equally high in all other investigated organs (Figure 4B). Liver immune phenotyping demonstrated higher levels of F4/80^+^ macrophages and lower levels of NK cells in Wildlings compared with SPF mice, but similar abundance of DC and neutrophils (Figure 4C; Figure S2A). Wildlings showed a slightly decreased percentage of CD8^+^ T cells in comparison to SPF mice in the liver, but equal total numbers of CD8^+^ T cells and LCMV‐GP33‐specific CTLs (Figure 4D). Interestingly, PKO‐Wildlings exhibited lower frequencies and absolute numbers of FoxP3^+^ CD25^+^ and Helios^+^ T_regs_ compared with PKO‐SPF mice in the liver (Figure 4D). Similar changes were also observed in the spleen (Figure S2B,C). Analysis of liver sections revealed a slight increase in hemophagocytic F4/80^+^ macrophages in PKO‐Wildling compared with PKO‐SPF mice (Figure 4E). In line with previous reports [35], liver infiltration of CD8^+^ lymphocytes was prominent in all PKO mice compared to B6‐SPF mice (Figure 4F), but CD8^+^ cell numbers per mm^2^ liver tissue were moderately lower in PKO‐Wildling than in PKO‐SPF mice. Thus, the presence of a natural microbiome in PKO‐Wildlings enhances immune‐mediated liver inflammation during LCMV‐induced primary HLH.

### 2.5 The Wildling Microbiome Modulates Immunological Features of LCMV‐Induced HLH

**FIGURE 5:**
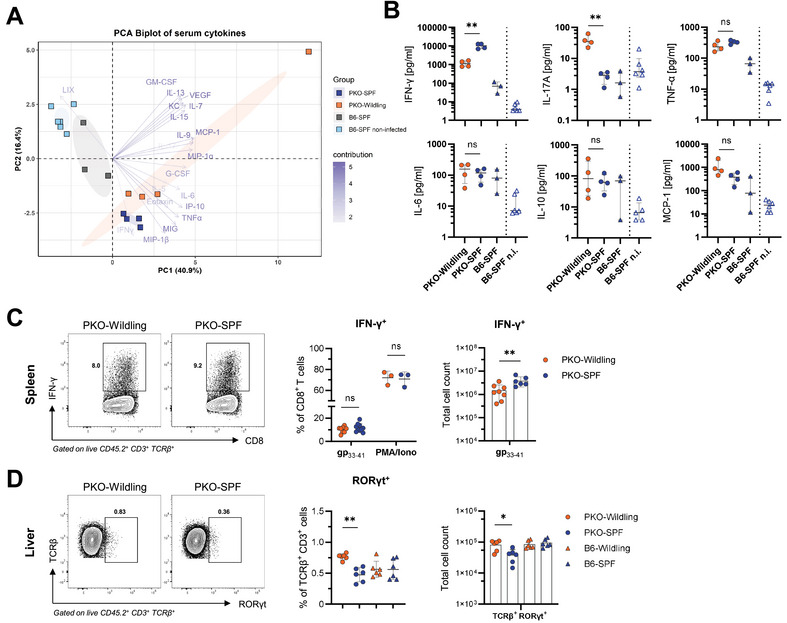
Wildling microbiota modulates the cytokine pattern in LCMV‐triggered HLH. (A) PCA Biplot of 32 cytokines analyzed by multiplex assay in serum of mice on day 12 after LCMV‐WE infection. Points represent individual mice and colored arrows indicate the contribution of selected cytokines. (B) Serum concentration of selected HLH‐related cytokines. Horizontal line and error bars indicate the median with interquartile range. (C) IFN‐γ expression of CD8^+^ T cells after ex vivo restimulation with GP_33‐41_ peptide (left). The abundance of IFN‐γ‐producing CD8^+^ T cells in spleens upon ex vivo restimulation with GP_33‐41_ peptide or PMA/Ionomycin (right). (D) RORγt expression in TCRβ^+^ T cells isolated from the liver (left). Frequency and absolute number of RORγt^+^ T cells in the liver (right). (C, D) Shown is mean ± SD. (A–D) Results shown are representative of two experiments performed with similar outcomes (A, B) or pooled from two independent experiments with 2–4 mice/group (C, D). Statistical testing was done by unpaired Student's *t*‐test (B‐D).

Organ immunopathology and progressive multiorgan failure in HLH result from excessive immune cell activation and hypercytokinemia [6]. To analyze the impact of the Wildling microbiome on the cytokine storm seen in HLH, we performed multiplex cytokine profiling of serum collected on day 12 following LCMV infection. Principal component analysis was applied to a set of 32 cytokines to identify patterns of cytokine abundance. Noninfected and LCMV‐infected B6‐SPF mice clustered separately from all PKO mice. In addition, PKO‐Wildling separated from PKO‐SPF mice (Figure 5A). Analysis of selected cytokines revealed a 10‐fold decrease in IFN‐γ, while IL‐17 was elevated in PKO‐Wildling compared with PKO‐SPF mice (Figure 5B). Other cytokines including TNF‐α, IL‐6, IL‐10, and macrophage chemoattractant protein‐1 (MCP‐1) were similar in PKO‐Wildling and PKO‐SPF mice (Figure 5B).

Given the reduced IFN‐γ and increased IL‐17 serum levels in PKO‐Wildlings, we examined intracellular IFN‐γ production of T cells and used RORγt expression as a surrogate for IL‐17 production to identify potential cellular sources contributing to this profile. Splenic CD8^+^ T cells from PKO‐Wildlings showed similar activation levels and IFN‐γ production upon ex vivo restimulation with GP_33‐41_ peptide or PMA/Ionomycin (Figure 5C, Figure S3A,B). However, the total number of IFN‐γ‐producing virus‐specific CTLs was lower in PKO‐Wildlings compared with PKO‐SPF mice (Figure 5C). In addition, we observed more RORγt^+^ TCRαβ^+^ T cells in the liver and mLN, but not in the spleen, of PKO‐Wildlings (Figure 5D, Figure 3C,D). In conclusion, the altered abundance of IFN‐γ and IL‐17‐producing cells in PKO‐Wildlings likely influences systemic cytokine levels, though other cell types may also contribute. This shifted cytokine profile may contribute to the observed changes in disease manifestations.

## 3 Discussion

The PKO mouse model has been fundamental in understanding the mechanisms driving HLH. However, it is increasingly recognized that the mouse model does not fully capture the human disease. This includes apparent differences in the immune threshold of disease initiation: While almost all patients with perforin null mutations present with disease in the first 6 months of life often without apparent infectious trigger [13], PKO mice held under SPF conditions remain healthy and develop HLH only after persistent infection with perforin‐controlled LCMV or MCMV, but not when infected with various other viruses, that are eliminated in the absence of perforin [11, 16, 21]. In this study, we tested whether the restricted SPF microbiome may be responsible for this difference. However, PKO‐Wildlings neither developed spontaneous HLH over a year of monitoring nor did they acquire higher HLH susceptibility as tested with systemic vaccinia virus infection. This suggests that the microbiome alone is insufficient to trigger HLH, and immune reactions elicited by “natural” immune stimulation are controlled independently of lymphocyte cytotoxicity in mice. Moreover, this study highlights that immune priming by microbiota does not allow the threshold of HLH induction to be reached by acute immune activation through a systemically spreading, but eventually eliminated viral infection.

It is well established that microbiome–host–immune crosstalk shapes innate and adaptive immunity and profoundly influences baseline immune activation [22, 23, 25]. Analysis of naïve PKO‐Wildlings and B6‐Wildlings revealed an increased abundance of effector/memory CD8^+^ T cells at steady state. These observations align with previous reports showing a similar enrichment of antigen‐experienced lymphocytes in B6‐Wildlings [30] and mice living under barrier‐free conditions [23, 27, 32]. Consistent with findings in animals with wild microbiome [27, 30, 32], PKO‐Wildlings had increased frequencies of CD4^+^ T_regs_ in the spleen and liver, which may help counteract the heightened basal immune activation. The increased abundance of TCRαβ^+^ T cells expressing RORγt indicates a rise in Th17 cells in Wildlings, as previously reported [30, 36]. In summary, Wildlings display an altered immune landscape in lymphatic organs at steady state, including elevated proportions of T cells, F4/80^+^ macrophages, and neutrophils. This sets the stage for altered responses to future pathogen encounters.

16S ribosomal RNA‐sequencing revealed similar microbial diversity and colonization in PKO‐Wildlings and B6‐Wildlings. This suggests that the microbiome was efficiently transferred by fostering and that the observed changes at steady‐state and after LCMV infection in PKO‐Wildlings are the consequence of early‐life microbial exposure. Notably, bacteria from the phylum Bacteroidetes, including segmented filamentous bacteria, were shown to play a role in the induction of local and systemic Th17 cells, while the frequency of Th17 cells is negatively correlated with Firmicutes in healthy individuals [37, 38]. Furthermore, studies in germ‐free and antibiotic‐treated animals corroborate a close association of both gut microbes and their metabolites with Th17 and T_reg_ cell differentiation [39]. Thus, the altered pattern of microbial colonialization likely accounts for the induction of T_regs_ and Th17 cells in Wildlings.

Infection of PKO‐Wildlings with LCMV resulted in HLH, which was comparable in severity to PKO‐SPF mice. In general, our results support the view that perforin‐mediated cytotoxicity is not a universal mechanism to limit virus‐induced inflammation but rather restricted to infections with specific viruses, such as LCMV, which are controlled by perforin. The unique ability of LCMV to induce HLH in mice is linked to its capacity to directly infect antigen‐presenting cells, allowing potent stimulation of a CTL response without the requirement of cross‐priming [40, 41]. Of note, while LCMV might be part of the full natural wild microbiota, its presence in our Wildling colony was excluded. Importantly, it has been described that low‐dose LCMV infection (5 pfu LCMV‐Armstrong i.p.) of PKO neonates does not lead to HLH‐associated mortality [42]. However, it remains unclear whether low‐titer LCMV transmission via breastfeeding and social grooming would induce HLH in PKO pups.

Although HLH parameters after LCMV infection were fulfilled in both PKO‐Wildling and PKO‐SPF mice, we observed differences in the pattern of HLH manifestations. We noted an increase in liver immunopathology, an altered cytokine signature, and less pronounced anemia in PKO‐Wildlings. Hepatomegaly is a frequent observation in HLH patients and can progress to severe cytokine‐mediated hepatic dysfunction without intervention [43]. Moreover, disruption of gut homeostasis and the proinflammatory cytokine IL‐17 play critical roles in the pathogenesis of inflammatory liver diseases [44]. Thus, enhanced liver damage in PKO‐Wildlings may be linked to the effects of the microbiome exerted on the liver via the gut–liver axis. Elevated levels of IL‐17, if acting on tissue level and in synergy with TNF‐α, might further fuel liver inflammation [45]. It has been proposed that IFN‐γ and T_regs_ suppress pathogenic IL‐17 responses, with IL‐17 serum levels positively correlating with liver damage in mouse hepatitis virus infection [46, 47]. The concurrent increase in serum IL‐17 and decrease of IFN‐γ levels in PKO‐Wildlings suggests a shift in the Type1/Type17 immune balance. This was also reflected by the increased abundance of RORγt^+^ TCRαβ^+^ T cells in the liver while IFN‐γ‐producing CD8^+^ T cells and NK cells were reduced. The decreased levels of IFN‐γ align well with the improved red blood cell counts observed in PKO‐Wildlings since anemia in HLH is an IFN‐γ‐dependent manifestation [48, 49]. In contrast, uncontrolled activation of macrophages exposed to inflammatory cytokines leads to IFN‐γ‐independent hemophagocytosis [48]. The increased abundance of F4/80^+^ CD11b^+^ and hemophagocytic macrophages in the livers of PKO‐Wildlings is another element contributing to increased liver inflammation. As reported earlier for primary HLH [50], we noted a collapse of T_regs_ in PKO‐SPF mice and PKO‐Wildlings after LCMV infection, which was stronger in the latter. In particular, PKO‐Wildlings showed diminished levels of the highly suppressive Helios^+^ T_regs_ [51]. This shifted balance of protective versus pathogenic cell populations may not only impact liver inflammation locally but also affect systemic immunity with consequences for the cytokine storm and the clinical manifestations of HLH.

In summary, this study provides the first evidence that the microbiome can influence HLH immunopathology by shifting the immune response polarization. It remains to be investigated how these results translate to humans and whether the microbiome is a factor shaping the clinical presentation of HLH in patients. Given the fact that microbial colonization is initiated with birth and major changes in the composition occur within the first years of life [52], it is tempting to speculate that such changes may impact the pattern of manifestations of primary HLH. In light of the results of this study, the microbiome might be one of several factors responsible for the disease variability observed in patients. Novel insights into the role of the microbiome in the context of primary HLH may be of interest with regard to therapy targeting specific cytokines.

## 4 Data Limitations and Perspectives

This study primarily focuses on the effect of the microbiota on HLH development in PKO mice. C57BL/6 mice were included as additional controls. For a direct comparison of PKO and wild‐type mice, future studies should consider the use of littermate controls for more robust comparisons. A complementary analysis of the immune landscape in other organs, including the intestinal tract, could offer deeper insights into microbiota‐controlled factors involved in HLH immunopathology. The impact of the microbiota on the immunological and clinical manifestations of HLH needs to be validated in human patients. The specific bacterial species and the mechanisms responsible for reshaping the immune landscape remain to be identified. The reason for the different thresholds for HLH‐induction in perforin‐deficient humans and mice remains unexplained. Overall, the contribution of the microbiome to shaping immune responses and disease manifestations in HLH and other hyperinflammatory diseases should be further investigated.

## 5 Materials and Methods

### 5.1 Mice

Mouse experiments were approved by the Regierungspräsidium Freiburg (G‐21/065). PKO (C57BL/6‐Prf1^tm1Sdz^) mice were obtained from Dr. Hengartner—University Hospital Zurich (JAX: #002407). B6 mice were purchased from Janvier, France (#SC‐C57N‐F) and maintained at the Center for Experimental Models and Transgenic Service (CEMT, Freiburg). Both lines were housed in individually ventilated cages under SPF conditions in a facility distinct from that of Wildlings (health report SPF facility: Table S1). For experiments, SPF mice were transferred to an experimental unit within another animal facility with similar hygiene standards.

PKO‐Wildlings were generated by fostering. Timed pregnant breeding pairs of B6‐Wildlings, generated through inverse germ‐free re‐derivation [26], and SPF‐housed PKO mice were set up. PKO‐SPF newborns were transferred to B6‐Wildling foster mothers immediately after birth and tail clipping was used to distinguish the pups. PKO‐Wildlings were maintained by homozygous inbreeding of fostered PKO mice. B6‐Wildlings and PKO‐Wildlings were kept under seminatural housing conditions as described earlier [30]. The presence of LCMV in the Wildling colony was excluded (health report Wildling facility: Table S2).

### 5.2 16S Ribosomal RNA Gene Sequencing

Sample preparation and raw data processing were performed by QIAGEN Genomic Services. Briefly, DNA was extracted from stool samples using the QIAamp PowerFecal Pro DNA kit. Libraries were prepared using the QIAseq 16S/ITS Region Panel, with target regions amplified by 12 PCR cycles. Sequencing adapters were added and enriched through an additional PCR. Libraries were cleaned, quality controlled, pooled at equimolar concentrations, and quantified by qPCR. Sequencing was performed on a NextSeq 2000 (Illumina) following the manufacturer's instructions. Raw data was de‐multiplexed using the QIAseq 16S/ITS Demultiplexer tool (CLC Genomics Workbench, version 23.0.5). After quality control and removal of regions with low reads (<100), operational taxonomic clustering for the 16S regions V1V2 was performed using the OTU clustering tool. Principle coordinate analysis was done using RStudio (version 2024.04.2+764) and the vegan package (version 2.6‐6.1). Graphs were created using ggplot2 (version 3.5.1).

### 5.3 Cell Isolation

Spleens and mesenteric lymph nodes were mechanically dissociated on a 100 µm cell strainer in RPMI containing 10% FCS, 1% penicillin/streptomycin, and 10 U/mL DNase I (Sigma, #DN25‐1G). Livers were mechanically dissociated with scissors and incubated in IMDM containing 1 mg/mL collagenase D (Sigma, #11088866001), 0.1 mg/mL DNase I (Sigma, #D4263), and 10 mM HEPES for 30 min at 37°C on a shaker (150 rpm). Afterwards, immune cells were purified using a 40%–60% Percoll density gradient centrifugation. Absolute cell counts were determined using a Neubauer counting chamber.

### 5.4 Infection Experiments

Nonrandomized, age‐matched male and female mice were infected i.v. with 200 pfu of lymphocytic choriomeningitis virus strain WE (originally obtained from Dr. Lehmann‐Grube, Hamburg, Germany) or 2 × 10^6^ pfu vaccinia virus strain WR. Investigators were not blinded. Body weight was monitored and mice were eliminated if they had lost ≥ 25% of their initial body weight or showed apathy or neurological failures. Ear temperature was measured with ThermoScan6 and 6022 (BRAUN). LCMV was quantified by focus‐forming assay on MC‐57 cells [53]. VV_WR_ titration and determination of viral load were performed by plaque assay on monolayer BSC‐40 cells [54]. BSC‐40 and MC57 cells were grown in DMEM supplemented with 10% fetal calf serum, 1% penicillin/streptomycin, and 1% l‐Glutamine.

### 5.5 Blood and Serum Analysis

Blood counts were determined with Sysmex KX‐21 hematology analyzer. Serum concentrations of GPT, lactate‐dehydrogenase, ferritin, and triglycerides were measured using Roche Modular Analytics Evo. sCD25 was analyzed by IL‐2Ralpha DuoSet kit (R&D Systems, #DY2438). Serum cytokines and chemokines were analyzed by Eve Technologies Corporation (Calgary, Canada) using a mouse 32‐plex discovery (MD32) immunoassay (Table S4). In addition, serum cytokines were measured using LEGENDplex Mouse Inflammation Panel (13‐plex, BioLegend, #740446). Principal component analysis and visualization were conducted using R (version 4.3.1) and packages FactoMineR (version 2.9) and factoextra (version 1.0.7).

### 5.6 Flow Cytometry

Antibodies were purchased from eBioscience, BD, and BioLegend (Table S3). Surface staining was performed ≥20 min at 4°C. For detection of myeloid cells, samples were pre‐incubated with anti‐CD16/CD32 (BD, #553142) for 10 min at 4°C. For live‐dead discrimination, Zombie NIR (BioLegend, #423106) was used. LCMV‐GP‐specific CD8^+^ T cells were stained using PE‐conjugated H‐2D^b^ MHC‐tetramers loaded with GP_33‐41_ (in‐house production and Tetramer Core Facility, Baylor College of Medicine) for 30 min at 4°C prior to surface staining. Detection of IFN‐γ after antigen‐specific restimulation with GP_33‐41_ peptide (1 µM) or PMA (50 ng/mL), and Ionomycin (1 µg/mL) for 4 h at 37°C was performed using Cytofix‐Cytoperm‐Kit (BD Biosciences, #554722) as described earlier [55]. Intranuclear staining of transcription factors was done using the FoxP3/Transcription Factor Staining Buffer Set (eBioscience, #00‐5523‐00). Samples were acquired on BD LSRFortessa and Sony ID7000 Full Spectral Analyzer and analyzed using FlowJo V10.

### 5.7 Liver Histology

Livers were fixed in 4% paraformaldehyde for up to 48 h. Paraffin sections were pre‐treated with citrate buffer for 30 min, blocked with 5% bovine serum albumin, goat serum, and Avidin/Biotin (Dako, #X0590). Sections were incubated overnight at 4°C with 1:200 rabbit anti‐mouse F4/80 (Cell Signaling, #70076) and biotinylated anti‐rabbit IgG (Dako, #E0432). For CD8^+^ staining, sections were pretreated with Dako Target Retrieval Solution (pH 9) for 20 min, blocked with 10% goat serum and Avidin/Biotin solution, and incubated with 1:200 rabbit anti‐mouse CD8a (Cell Signaling, #98941S) and biotinylated anti‐rabbit IgG overnight. F4/80^+^ and CD8^+^ cells were visualized using the Dako REAL Detection System, alkaline phosphatase/RED, and hematoxylin counter‐staining. Hemophagocytosis was quantified by counting 10 high‐power fields (40×) per organ. Images were captured using a Carl Zeiss AxioImager at 10× or 40× magnification and acquired with AxioVision software.

### 5.8 Statistical Analysis

Statistical testing was performed using GraphPadPrism 9 and 10 (GraphPad Software, San Diego, California, USA). Details on statistical testing are reported in the figure legend. Asterisks indicate statistical significance (^∗^ *p* < 0.05; ^∗∗^ *p* < 0.01; ^∗∗∗^ *p* < 0.001, ^****^ *p* < 0.0001).

## Author Contributions

Jasmin Mann: Conceptualization, formal analysis, validation, investigation, methodology, visualization, data curation: writing–original draft. Solveig Runge: Methodology, animal fostering, investigation. Christoph Schell and Katja Gräwe: Histological analysis, validation, visualization. Gudrun Thoulass, Jessica Lao, Sandra Ammann, Sarah Grün, Christoph König, Sarah A. Berger, Benedikt Hild: Formal analysis, investigation. Peter Aichele: Conceptualization, formal analysis, methodology, investigation. Stephan P. Rosshart: Conceptualization, methodology, investigation, funding acquisition. Stephan Ehl: Conceptualization, data curation, investigation, project administration, funding acquisition, supervision, writing–original draft. All authors contributed in writing–review and editing.

## Conflicts of Interest

The authors declare no conflicts of interest.

### Peer Review

The peer review history for this article is available at https://publons.com/publon/10.1002/eji.202451061.

## Supporting information



Supporting Information

## Data Availability

This study includes no data deposited in external repositories.
