# Supplementary material for: The Microbiome Modifies Manifestations of Hemophagocytic Lymphohistiocytosis in Perforin‐Deficient Mice
Source: Eur J Immunol. 2024 Nov 16;55(1):e202451061. doi: 10.1002/eji.202451061 (PMC11739664; doi:10.1002/eji.202451061)
Supplement: Supplementary file 1 — Supporting Information [file EJI-55-e202451061-s001.pdf]

Supporting information - Suppl. Figure 1

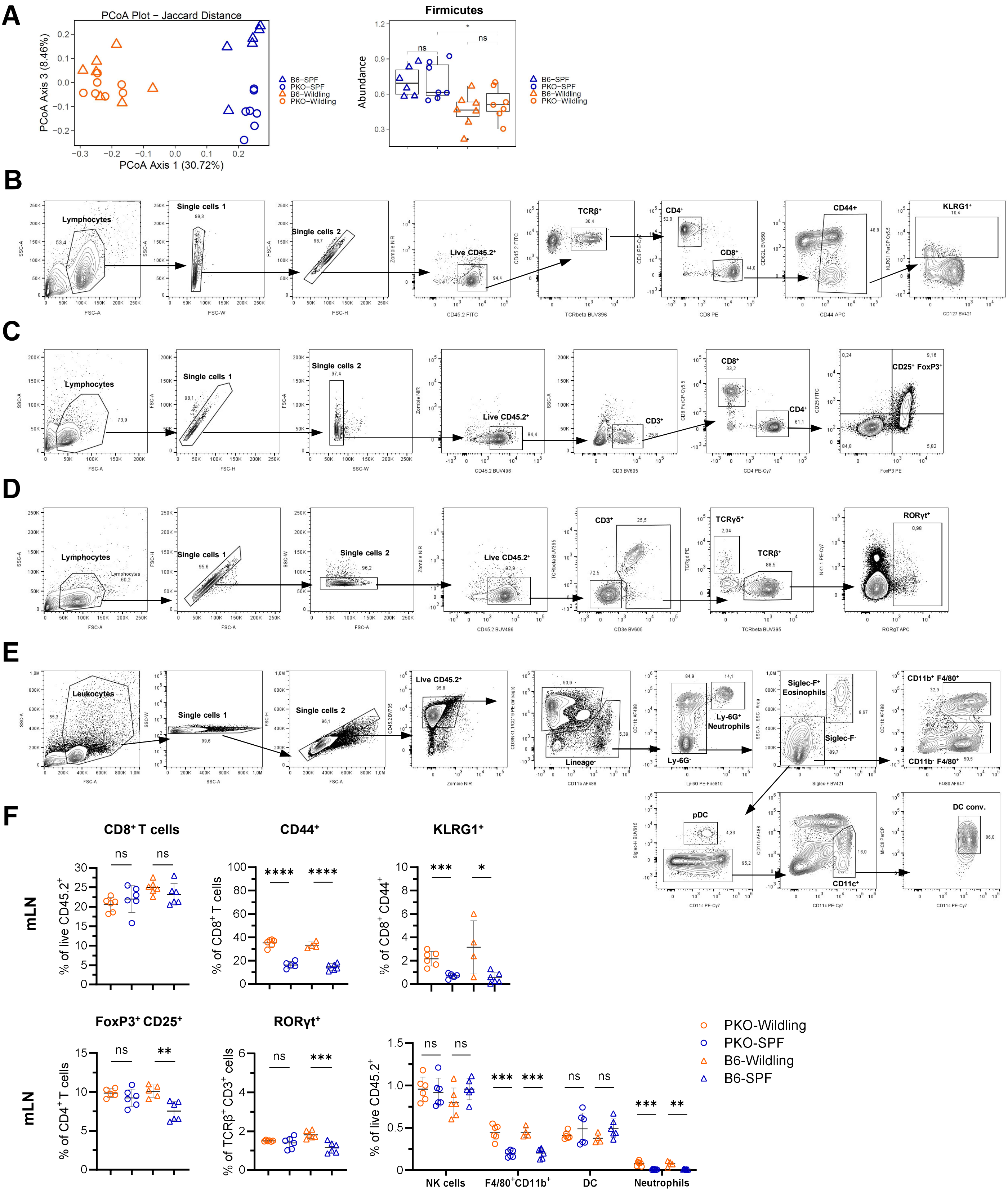

Suppl. Fig. 1: A Principal coordinate analysis of 16S ribosomal RNA profiling data using principal coordinates 1 and 3 (left) and abundance of Firmicutes in fecal samples of Wildings and SPF mice (right). Symbols represent mice. **B** Representative gating strategy for analysis of antigen-experienced effector/memory CD8$^+$ T cells (CD44$^+$) and KLRG1-expressing cells among gated CD44$^+$ CD8$^+$ T cells. **C** Gating strategy to identify FoxP3$^+$ CD25$^+$ T$_{regs}$. **D** Contour plots show the gating strategy for analysis of RORγt$^+$ TCRαβ$^+$ T cells. **E** Gating strategy to analyze CD11b$^+$ F4/80$^+$ macrophages, Ly-6G$^+$ neutrophils and dendritic cells (DC = pDC + DC conv. gate). **F** Frequency of indicated populations in mesenteric lymph nodes (mLN). Data represent mean ± SD, pooled from two independent experiments with 3 mice per group. Statistical significance was determined using unpaired Student's t test or multiple unpaired t tests with multiple comparisons correction performed using the Holm-Šidák method.

Supporting information - Suppl. Figure 2

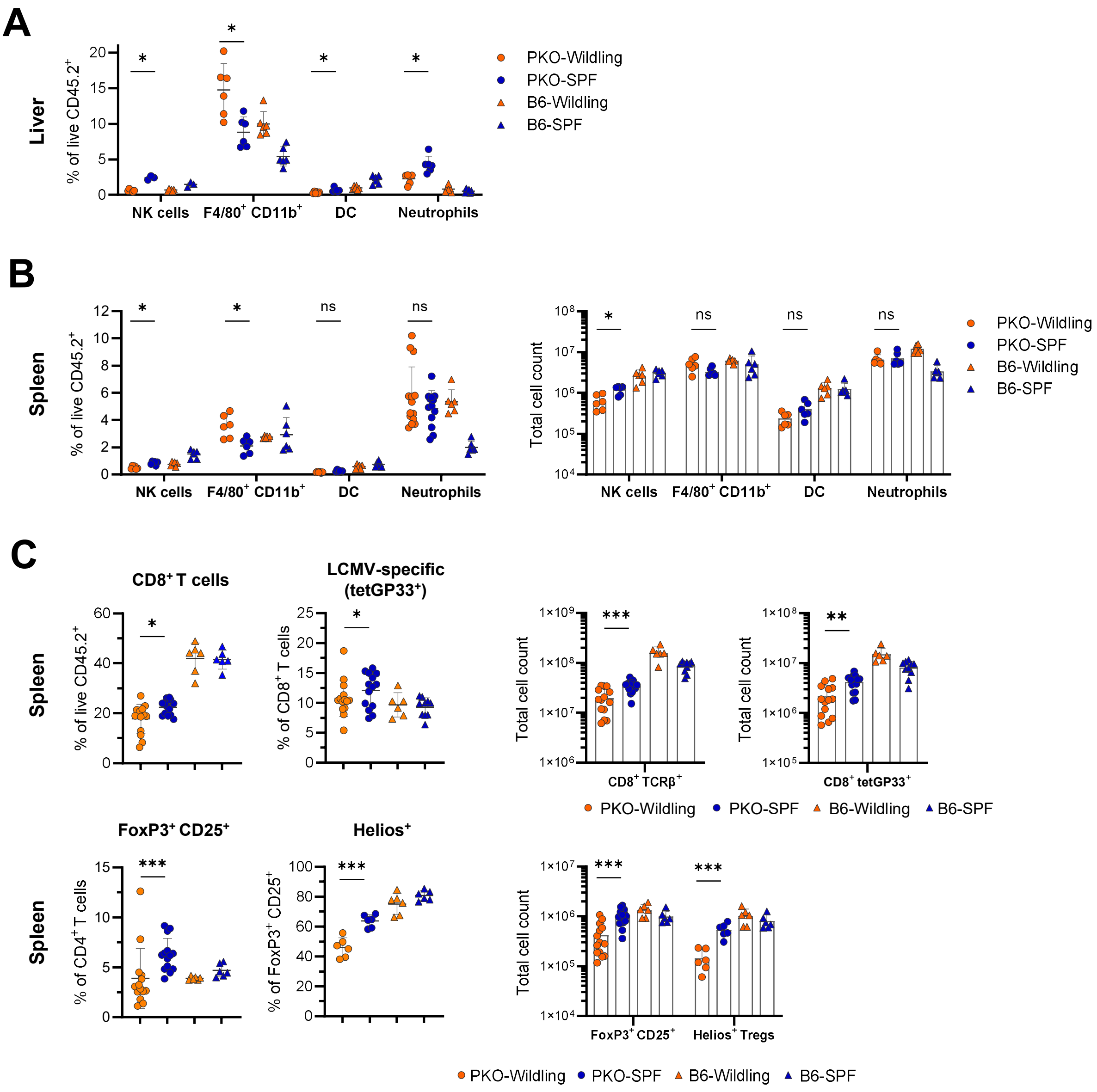

Suppl. Fig. 2: A Percentage of indicated cell populations in the liver of LCMV-infected mice analyzed on day 11/12 after infection. **B** Frequency and absolute numbers of indicated populations in the spleen of LCMV-infected mice. **C** Frequency and absolute number of CD8⁺, tetGP33⁺ CD8⁺ T cells, FoxP3⁺ CD25⁺ T_{regs} and Helios⁺ T_{regs} in the spleen. **A-B:** Data shows mean ± SD, pooled from at least 2 independent experiments with 3 mice/group. Statistical testing was performed using multiple unpaired t tests with correction for multiple comparison using the Holm-Šidák method (A,B), and Mann-Whitney test or unpaired Student's t test (C).

Supporting information - Suppl. Figure 3

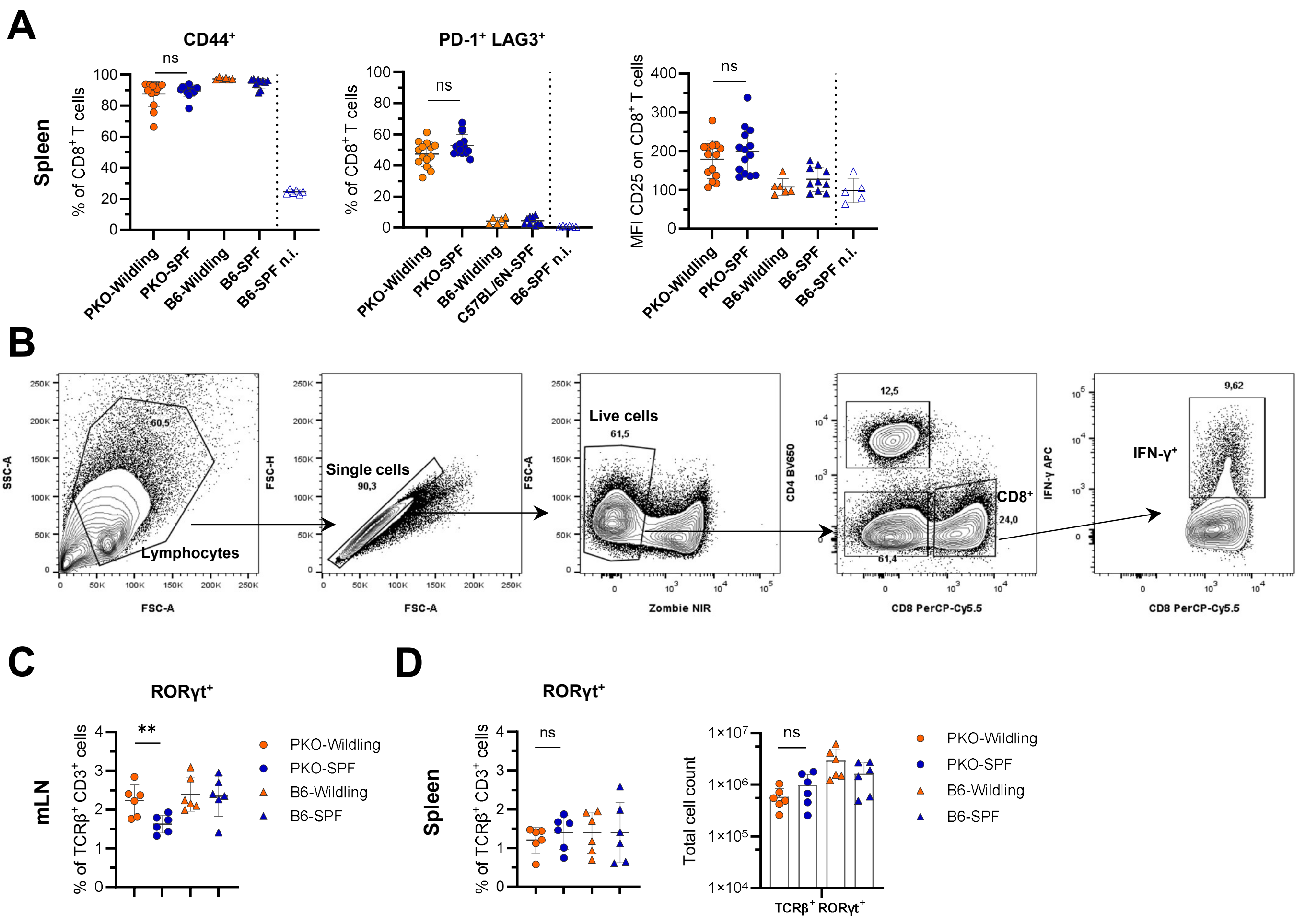

Suppl. Fig. 3: **A** Percentage of CD44⁺, PD-1⁺ and LAG-3⁺ cells among CD8⁺ T cells and median fluorescence intensity (MFI) of CD25 on splenic CD8⁺ T cells on day 11/12 after LCMV infection. **B** Gating strategy for intracellular IFN-γ staining of isolated splenocytes after 4h *ex vivo* restimulation. **C** Frequency of RORγt-expressing cells in mesenteric lymph nodes (mLN) on day 12 after LCMV infection. **D** Relative and absolute abundance of RORγt-expressing cells in spleen of LCMV-infected mice. **A,C-D:** Data represents mean ± SD, pooled from at least 2 independent experiments with 3 mice/group. Statistical testing was performed using unpaired Student's t test.

Supporting information

Supplementary Table S1: Health report for SPF colony. Test results were obtained by serology or PCR between March 2021 and March 2023.

**Viruses**	**Present within the colony**
Ectromelia virus	No
Mouse Rotavirus	No
Hantaan	No
K Virus/Murine Pneumotropic Virus	No
Lymphocytic choriomeningitis virus (LCMV)	No
Mouse Adenovirus type (MAV) 1 & 2	No
Mouse Hepatitis Virus (MHV)	No
Mouse Kidney Parvovirus (MKPV)	No
Mouse Parvovirus (MPV)	No
Minute Virus of mice (MVM)	No
Pneumonia Virus of Mice (PVM)	No
Murine Norovirus (MNV)	**Yes**
Reovirus type 3 (REO3)	No
Sendai virus	No
Theiler's murine encephalomyelitis virus (TMEV)	No
**Bacteria, Mycoplasma and Fungi**	**Present within the colony**
Pasteurella pneumotropica	No
Pasteurella pneumotropica biotype Heyl	**Yes**
Pasteurella pneumotropica biotype Jawetz	**Yes**
Proteus mirabilis	No
Pseudomonas aeruginosa	No
Rodentibacter heylii	**Yes**
Rodentibacter pneumotropicus	No
Salmonella species	No
Staphylococcus aureus	**Yes**
Streptobacillus moniliformis	No
Streptococcus pneumoniae	No
Streptococcus sp. β-hemolytic Group A	No
Streptococcus sp. β-hemolytic Group B	No
Streptococcus sp. β-hemolytic Group C	No
Streptococcus sp. β-hemolytic Group G	No
Bordetella bronchiseptica	No
Citrobacter rodentium	No
Clostridium piliforme	No
Corynebacterium kutscheri	No
Helicobacter species	**Yes**
Helicobacter bilis	No
Helicobacter ganmani	No
Helicobacter hepaticus	No

Helicobacter mastomyrinus	No
Helicobacter rodentium	**Yes**
Helicobacter typhlonius	No
Klebsiella oxytoca	**Yes**
Klebsiella pneumonia	No
Mycoplasma pulmonis	No
**Parasites and Protozoa**	**Present within the colony**
Chilomastix species	No
Cryptosporidium species	No
Eimeria species	No
Entamoeba muris	**Yes**
Giardia muris	No
Mite species	No
Myocoptes	No
Radfordia/Myobia	No
Spironucleus muris	No
Pinworm species	No
Aspiculuris tetraptera	No
Syphacia muris	No
Syphacia obvelata	No
Tritrichomonas muris	No

Supporting information

Supplementary Table S2: Health report for Wildling colony. Presence of indicated microbes was tested by serology or PCR from fecal pellets and fur swabs from Wildling animals between June 2021 and March 2022.

**Viruses**	**Present within the colony**
Ectromelia virus	No
Hantavirus	No
K Virus/Murine Pneumotropic Virus	No
Lymphocytic choriomeningitis virus (LCMV)	No
Minute Virus of Mice (MVM)	No
Mouse Adenovirus type (MAV) 1 & 2	No
Mouse Hepatitis Virus/Murine coronavirus (MHV)	No
Mouse Kidney Parvovirus (MKPV)	No
Mouse Parvovirus (MPV)	**Yes**
Mouse Rotavirus	No
Mouse Thymic Virus	No
Murine Norovirus (MNV)	**Yes**
Pneumonia Virus of Mice (PVM)	No
Reovirus type 3 (REO3)	No
Sendai virus	No
Theiler’s murine encephalomyelitis virus (TMEV)	**Yes**
**Bacteria**	**Present within the colony**
Klebsiella oxytoca	No
Klebsiella pneumoniae	No
Bordetella bronchiseptica	No
Citrobacter rodentium	No
Clostridium piliforme	No
Corynebacterium kutscheri	No
Helicobacter species	**Yes**
Helicobacter bilis	No
Helicobacter ganmani	**Yes**
Helicobacter hepaticus	No
Helicobacter mastomyrinus	**Yes**
Helicobacter rodentium	No
Helicobacter typhlonius	**Yes**
Mycoplasma pulmonis	No
Rodentibacter pneumotropicus	**Yes**
Rodentibacter heylii	**Yes**
Proteus mirabilis	No
Pseudomonas aeruginosa	**Yes**
Salmonella species	No
Staphylococcus aureus	No
Streptobacillus moniliformis	No

Streptococcus pneumoniae	No
Streptococcus sp. β-hemolytic Group A	No
Streptococcus sp. β-hemolytic Group B	No
Streptococcus sp. β-hemolytic Group C	No
Streptococcus sp. β-hemolytic Group G	No
**Parasites/Protozoa/Fungi**	**Present within the colony**
Cryptosporidium species	**Yes**
Entamoeba muris	**Yes**
Giardia muris	**Yes**
Eimeria species	No
Chilomastix species	**Yes**
Spironucleus muris	**Yes**
Tritrichomonas muris	**Yes**
Pinworm species	**Yes**
Aspiculuris tetraptera	**Yes**
Syphacia muris	No
Syphacia obvelata	**Yes**
Mite species	**Yes**
Myocoptes	**Yes**
Radfordia/Myobia	**Yes**

Supporting information

Supplementary Table S3: Antibodies used for flow cytometry.

Target antigen	Fluorochrome	Clone	Dilution	Company	Cat#
CD45.2	BV785/AF488	104	100/200	BioLegend	109839/109816
CD45.2	BUV496	104	100	BD Horizon	569670
CD3ε	PE-Cy7/BV605	145-2C11	300/100	BioLegend	100320/100351
CD3ε	PE	17A2	100	BioLegend	100206
CD19	PE	1D3/CD19	200	BioLegend	152407
NK1.1	PE/PE-Cy7	PK136	100/200	BioLegend	108708/108714
TCR β chain	BUV395	H57-597	100	BD Horizon	569248
γδ TCR	PE	GL3	100	BD Pharmingen	553178
CD8a	BV510/PerCP-Cy5.5	53-6.7	200/400	BioLegend	100752/100734
CD8a	PE	53-6.7	100	eBioscience	12-0081-83
CD4	APC/APC-Fire750/BV650	RM4-5	500/100/1000	BioLegend	100516/116020/100555
CD4	PerCP-Cy5.5	GK1.5	200	BioLegend	100434
CD4	PE-Cy7	GK1.5	1000	eBioscience	100434
CD25	AF488	PC61	50	BioLegend	102017
FoxP3	eFluor450	FJK-16s	500	eBioscience	48-5773-82
Helios	eFluor450	22F6	100	eBioscience	48-9883-42
I-A/I-E (MHCII)	PerCP	M5/114.15.2	200	BioLegend	107623

CD11c	PE-Cy7	N418	100	BioLegend	117318
F4/80	AF647	BM8	100	BioLegend	123122
Ly-6G	PE-Fire810	1A8	400	BioLegend	127673
CD170 (Siglec-F)	BV421	S17007L	200	BioLegend	155509
Siglec-H	BUV615	440c	400	BD OptiBuild	751024
CD11b	AF488	M1/70	100	BioLegend	101217
CD11c	PE-Cy7	N418	100	BioLegend	117318
RORγ(t)	APC	B2D	100	eBioscience	17-6981-80
KLRG1	PerCP-Cy5.5	2F1	150	BioLegend	138418
KLRG1	APC	2F1	100	eBioscience	17-5893-82
CD127	BV421	A7R34	100	BioLegend	135027
CD44	APC/FITC	IM7	1500/200	eBioscience	17-0441-82/11-0441-85
CD62L	BV650	MEL-14	1500	BioLegend	104453
PD-1 (CD279)	BV785	29F.1A12	300	BioLegend	135225
PD-1 (CD279)	PerCP-eFluor710	J43	400	eBioscience	46-9985-82
LAG3 (CD223)	PE-Cy7	C9B7W	100	BioLegend	125226
IFN-γ	APC	XMG1.2	250	eBioscience/Invitrogen	17-7311-82
CD16/CD32 (Mouse BD Fc Block)	-	2.4G2	1 µg for 1x 10^6 cells	BD Pharmingen	553141

Supporting information

Supplementary Table S4: Cytokines/Chemokines analyzed in the Mouse Cytokine/Chemokine 32-Plex Discovery Assay® Array (MD32) by Eve technologies (Calgary, Canada).

**Cytokines/Chemokines**
Eotaxin
G-CSF
GM-CSF
IFNγ
IL-1α
IL-1β
IL-2
IL-3
IL-4
IL-5
IL-6
IL-7
IL-9
IL-10
IL-12p40
IL-12p70
IL-13
IL-15
IL-17A
IP-10
KC
LIF
LIX
MCP-1
M-CSF
MIG
MIP-1α
MIP-1β
MIP-2
RANTES
TNFα
VEGF-A
